# Improving medication dispensing and counselling for patients with vision impairment: a qualitative study of pharmacist-reported barriers and facilitators

**DOI:** 10.1186/s12913-024-11009-9

**Published:** 2024-04-26

**Authors:** Basma Y. Kentab, Heather E. Barry, Sinaa A. Al-Aqeel, Carmel M. Hughes

**Affiliations:** 1 Primary Care Research Group, School of Pharmacy, Queen’s University Belfast, 97 Lisburn Road, Belfast, Northern Ireland BT9 7BL UK; 2 Department of Clinical Pharmacy, College of Pharmacy, King Saud University, Riyadh, Saudi Arabia

**Keywords:** Qualitative research, Theoretical domains framework, Behaviour change, Vision impairment, Pharmacists, Dispensing, Counselling, Medicines optimisation

## Abstract

**Background:**

People with vision impairment encounter many difficulties when it comes to medicines use. However, evidence indicates that there are major gaps in pharmaceutical care service provision worldwide and limited research on interventions to optimise medication use for this patient population. The Theoretical Domains Framework (TDF) provides a method for theoretically understanding individuals’ behaviour and informing development of interventions. The aim of this research was to (a) identify the barriers and facilitators to the provision of medication dispensing and counselling services by pharmacists to patients with vision impairment, and (b) identify key TDF domains to be targeted in a future intervention.

**Methods:**

Semi-structured interviews were conducted with pharmacists from different pharmacy practice settings/areas in Saudi Arabia. The 14-domain TDF was utilised as the theoretical lens through which pharmacists’ behaviours were examined. Interviews were conducted in Arabic or English, either face-to-face or over the telephone based on the participant’s preference. Following transcription, interviews conducted in Arabic were translated into English before analysis. Data analysis involved using the framework method and content analysis to identify important barriers and facilitators to the provision of dispensing and counselling services to those with vision impairment. Key TDF domains that could be targeted in a future intervention were then identified using a consensus-based approach.

**Results:**

Twenty-six pharmacists were interviewed**.** Pharmacists’ experience in pharmacy practice ranged from two to 28 years. A range of barriers and facilitators were highlighted as important in providing services to those with vision impairment. Eight domains were identified as ‘key domains’ including: ‘Knowledge’, ‘Skills’, ‘Beliefs about capabilities’, ‘Goals’, ‘Memory, attention, and decision processes’, ‘Environmental context and resources’, ‘Social influences’, and ‘Behavioural regulation’.

**Conclusions:**

Barriers and facilitators identified by pharmacists will inform the development of an intervention to ensure its applicability to everyday practice. Future research will focus on the process of developing the proposed intervention through targeting key TDF domains to improve medication dispensing and counselling by pharmacists to patients with vision impairment.

**Supplementary Information:**

The online version contains supplementary material available at 10.1186/s12913-024-11009-9.

## Background

Pharmacists are more accessible than other healthcare professionals [1] and are also typically the last healthcare professionals who interact with patients before they take their medicines. However, reports suggest that many gaps exist in the delivery of pharmaceutical services by pharmacists to visually impaired patients and that routine services to support this population may not be available [2–5]. For example, issues have been reported with identifying patients with vision impairment, reliance on caregivers and family members, difficulties in communication with these patients, and organisational barriers (e.g. time constraints, workload, lack of access to patients’ records) that affected the provision of pharmaceutical care [3–5]. The reasons why pharmaceutical services are not routinely provided by pharmacists as part of normal practice are under-researched. There is also a paucity of interventions delivered by pharmacists aiming to support medicines optimisation in this patient population [6].

Helping patients to ‘take their medicines correctly, improve outcomes related to medicines use, and improve medicines safety’ are some of the essential goals of medicines optimisation [7]. The initial engagement between pharmacists and patients typically occurs when prescriptions are dispensed, and counselling is provided. This should ideally support patients to take medications appropriately and ultimately optimise their medication use. The provision of accessible medicines information by pharmacists when dispensing and counselling to patients with vision impairment has been identified as one of the issues that warrants further research [3–5, 8]. Other issues that require further attention include strategies to encourage disclosure or identification of vision impairment, the educational and training needs of pharmacists, and the development of effective interventions to support this patient population [3, 4, 8].

It is now recognised that to increase the likelihood of an intervention being effective, it is best practice to develop interventions systematically using the best available evidence and appropriate theory [9]. The use of theory helps with identifying the determinants (i.e. barriers and facilitators) of change that can then be targeted by an intervention [10–12]. While pharmacists’ views in relation to the provision of pharmaceutical services to patients with vision impairment have been explored in studies that have utilised quantitative [2, 5] and qualitative [4, 13] approaches, none of these relied on a theoretical underpinning to explain pharmacists’ behaviours and practices.

The Theoretical Domains Framework (TDF), which is an integrative theoretical framework, has provided a method for theoretically assessing professional and other health-related behaviours as a basis for intervention development [14]. The TDF comprises 128 theoretical constructs that were clustered in 12 domains [15] and was later refined to include 14 domains [16]. Being derived from 33 different behaviour and behaviour change theories [15], the wide-encompassing nature of the model makes it an appealing option to explore a wide range of determinants of behaviours. In addition, there has been extensive work around how TDF domains can be linked to BCTs and integrated into the development of behaviour change interventions [17–20]. Employing the TDF as the theoretical lens through which pharmacists’ behaviours are examined will aid in understanding the factors that influence the dispensing and counselling processes for visually impaired patients and provide the theoretical basis for designing an effective medicines optimisation intervention in this patient group.

The study reported here was part of a larger research project that took place in Saudi Arabia and aimed to develop an intervention to support the processes of dispensing and counselling for patients with vision impairment using the TDF as the theoretical framework. The project was developed in line with the United Kingdom’s (UK) Medical Research Council (MRC) guidance which advocates a systematic approach to developing interventions using the best available evidence and appropriate theory [9, 21]. Theoretical understanding of a behaviour is an integral part of developing and implementing a successful intervention and can be achieved by examining existing evidence and theory and undertaking primary research such as interviews with stakeholders [21]. The specific objectives of the current study were as follows:

- To utilise the TDF to identify the barriers and facilitators to medication dispensing and counselling from the viewpoint of pharmacists; and
- To identify the key TDF domains to be targeted through an intervention.

## Methods

The study adopted qualitative methodology through the use of semi-structured interviews. Since little is known about how pharmacists interact with visually impaired patients in Saudi Arabia, we needed a more in-depth understanding of real-life practices of pharmacists and whether there were differences between different pharmacy practice areas and settings. Knowing the long working hours of pharmacists and the range of settings in which they worked, it would have been extremely difficult to arrange focus groups and have pharmacists meet at an exact time and place. This made interviews a more realistic option to explore pharmacists’ experiences and views. This study is reported in line with the Consolidated criteria for reporting qualitative research (COREQ) checklist [22] (Additional file 1).

### Sampling and recruitment strategy

**Fig. 1:**
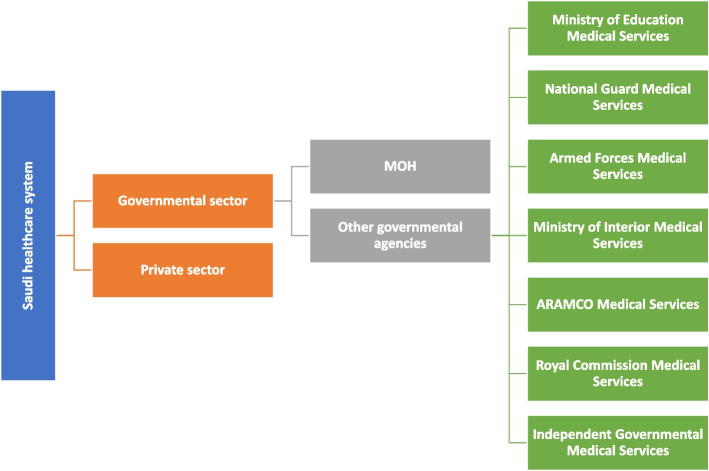
The Saudi healthcare system

Considering the unique Saudi health system [23, 24] (Fig. 1), a purposive sampling technique was utilised to ensure the inclusion of pharmacists from different practice areas (hospital and community) and different settings in Saudi Arabia [e.g. governmental hospitals and private hospitals; Ministry of Health (MOH) and Ministry of Education hospitals and chain and independent community pharmacies]. This was combined with snowball sampling.

Pharmacists with a minimum of one year’s experience working at a range of hospitals, or at independent/chain community pharmacies were eligible to participate in the study. Pharmacists had to be involved with performing dispensing and/or counselling to patients or with overseeing these processes, e.g. supervisors or managers.

Pharmacists were recruited through several approaches including an email through the Saudi Pharmaceutical Society (SPS), direct personal contact followed by an invitation letter sent to contact persons at the company headquarters of a number of Saudi Arabia's major chain community pharmacies, searching independent community pharmacy listings in the main districts of Riyadh city (East, West, South, North, Centre), and contacting pharmacy directors or personal contacts at target hospitals to identify pharmacists from practice areas that were found not to be represented in the sample after using the other approaches.

Eligible pharmacists were contacted by telephone to explain the research project and then sent an invitation letter and an information sheet via email or WhatsApp messages to provide further information. A follow-up call or email was made by the researcher one week later to ascertain the pharmacist’s interest in participating. If the pharmacist agreed to participate, a suitable time was arranged to conduct the interview. Recruitment and interviewing continued until theoretical saturation was reached, i.e. until no significant new concepts emerged from the analysis of the interviews.

### Topic guide development and data collection

Semi-structured interviews were conducted using an interview guide (Additional file 2) that was constructed based on the 14-domain TDF 2 [16] and informed by similar medicines optimisation research [25, 26]. There were structured questions to collect some background data as well as open-ended questions to explore each of the TDF domains. Additionally, prompts were incorporated to obtain additional information from participants as needed. Interviews began by providing definitions of vision impairment and medicines optimisation to help explain what they meant in the context of this study. The draft interview guide was piloted with three hospital outpatient pharmacists and minor changes in wording were made based on their feedback.

Pharmacist interviews were conducted either face-to-face at their place of work, or over the telephone based on the participant’s preference and/or location (i.e. inside/outside Riyadh city). Video conferencing was not widely utilised at the time of planning and conduct of this study. Telephone interviews were the best available option when face-to-face interviews could not be carried out. The researcher (BK), who is a native Arabic speaker and fluent in English, conducted all interviews in either English or Arabic (based on the preference of the participant). All interviews were recorded on a digital voice recorder and each interview was transcribed verbatim, with all identifiers removed and replaced with codes to differentiate between participants, e.g. CP01 (Community Pharmacist 1), or HP01 (Hospital Pharmacist 1). Interviews conducted in Arabic were translated into English by a professional translator. One full interview translation was reviewed by both BK and SA to ensure accuracy. BK reviewed all remaining translated transcripts for accuracy and any necessary corrections were made.

### Data analysis

Analysis was carried out through two sequential stages:

- Stage 1: Identifying barriers and facilitators to medicine dispensing and counselling

The framework method was used to deductively analyse the data through seven stages: transcription, familiarisation, coding, developing a working analytical framework, applying the analytical framework, charting data into the framework matrix and finally interpreting the data [27]. Initially, three transcripts were independently coded by both BK and another member of the research team. Coding was then discussed by the research team to compare and refine codes and agree on the final framework. BK subsequently carried out the coding of the remainder of the interviews. The data were managed using NVivo® 12 [28] before being imported into a Microsoft Excel spreadsheet to generate a framework matrix. The 14 TDF domains served as the coding labels [16]. Content analysis was also conducted to identify the barriers and facilitators of medicines dispensing and counselling within each TDF domain from the perspectives of pharmacists.

- Stage 2: Identifying key TDF domains to be targeted by an intervention

This process was undertaken by judging the relevance/importance of domains using three criteria: relatively high frequency of specific themes; presence of conflicting themes; and evidence of strong themes that may affect the target behaviour [14]. The feasibility of targeting barriers and facilitators as part of a future intervention within the hospital/community pharmacy setting, available resources, and the project timeframe guided the identification of key TDF domains. Decisions were made using a consensus-based approach within the research team.

## Results

### Participant characteristics

All pharmacists who were invited to participate agreed to be interviewed. Twenty-six pharmacists were interviewed between May 2019 and February 2020. Data saturation occurred at interview number 26 as no significant new data emerged at that point. Most interviews took place face-to-face at the pharmacist’s place of work (*n* = 19), while seven interviews were conducted over the telephone. Duration of interviews ranged from approximately 39 min to 140 min (median: 53, IQR: 28.5). The majority of pharmacists preferred to communicate in Arabic with technical phrases relevant to pharmacy expressed in English. Pharmacists’ experience in pharmacy practice ranged from two to 28 years (mean: 11.5, SD: 7.3). Most pharmacists worked in Riyadh city (*n* = 22), while three pharmacists worked in Jeddah (Western Province) and one pharmacist worked in Najran (Southern Province). Four pharmacists were working in the hospital outpatient pharmacies of two governmental hospitals that specialise in eye diseases. No pharmacists were interviewed from pharmacies in private hospital settings. Table 1 shows other demographic characteristics of participating pharmacists.

**Table 1:** Demographic information relating to pharmacist participants (*n* = 26)

Demographic characteristic	Number of respondents in each category (%)
**Gender**	
Female	8 (30.8)
Male	18 (69.2)
**Pharmacy setting**	
Community pharmacy	9 (34.6)
Chain pharmacy	7 (26.9)
Independent pharmacy	2 (7.7)
Governmental hospital pharmacy	17 (65.4)
Ministry of Health hospitals	6 (23.1)
Ministry of Education hospitals	4 (15.4)
Military hospitals	3 (11.5)
Independent hospitals	4 (15.4)
**Pharmacy practice area**	
Community pharmacy	9 (34.6)
Hospital outpatient pharmacy	9 (34.6)
Clinical pharmacy*	3 (11.5)
Managerial/supervisory role	5 (19.2)

*Clinical pharmacy: In Saudi Arabia, clinical pharmacists typically work in hospital settings to perform roles such as providing evidence-based medication recommendations, providing counselling to patients and their families, and offering medication information to other health care providers. Clinical pharmacists are required to obtain a postgraduate degree or residency training to practise as such [29]

### Identification of barriers and facilitators that influence medication dispensing and counselling to patients with vision impairment

Pharmacists identified many barriers and facilitators under each of the TDF domains. It was clear throughout the analytical process that domains overlapped, and many barriers and facilitators could be coded under more than one domain. For example, having the relevant knowledge and training was coded as a facilitator under the ‘Knowledge’ and ‘Skills’ domains as well as the ‘Beliefs about capabilities’ domain. Workforce shortage coded as a barrier under the ‘Environmental context and resources (ECR)’ domain was also identified as a barrier under the ‘Optimism’ domain.

The following is a summary of the important factors within each domain that were perceived to influence the provision of medication dispensing and counselling to visually impaired patients. An expanded version of the barriers and facilitators under each of the TDF domains, together with illustrative quotes can be found in Additional file 3.

#### Domain: Knowledge

Pharmacists identified knowledge of the presence of vision impairment in the patient as an important facilitator and reported various methods for identifying patients when they presented to them. Methods ranged from noticing the appearance of the patients and how they behaved, recognising certain diagnoses and comorbidities, to patients showing ‘disability cards’ indicating they were visually impaired.

*"Umm I mean this is the most important thing, to know that he has visual impairment."* (CP06)

Pharmacists reported that patients did not usually disclose that they had vision impairment unless they required assistance such as needing the pharmacist to write in a bigger font or to prioritise them in delivery of services as was the case with the ‘disability card’. Some pharmacists avoided explicitly questioning patients about vision impairment because they felt it was sensitive information and they were concerned about embarrassing patients. Pharmacists also indicated that they had no way of knowing that the patient was visually impaired if the caregiver came to collect medications.

*"No, no one mentions something like that. I don't think that he even likes disclosing it to someone. [...] So, I feel they don’t tell out of embarrassment, so I avoid stating it. I mean I deal with him based on what I see in front of me and that is it…or what I felt from him."* (CP04)

All pharmacists were unaware of the availability of guidance or advice on how to provide services to patients with vision impairment, and none had received any type of education about the issue at pharmacy schools.

"I mean, it would be easier for me if there were a guideline." (HP05).

#### Domain: Skills

Pharmacists listed a wide range of skills they believed were important when providing medication dispensing and counselling to patients with vision impairment including patience, communication skills, counselling skills (e.g. listening, simplifying language, describing shapes of tablets) and problem-solving skills. Although none of the pharmacists reported receiving any training on the issue, they acknowledged the importance of future courses and training in order to improve their skills when interacting with visually impaired patients. Some pharmacists attributed the lack of training to the low number of visually impaired patients seen in their practice setting.

*"I think communication is the most important thing. That you should not be, like, annoyed with the person in front of you, or appreciate his condition. So, you should be a little patient."* (CP04)

*"…if you are in an institution, umm, where you probably see many patients, […] then in this case umm, ok, …there should be training, there should be… using all tools that might…that might help. But for…let’s say at* [name of hospital where pharmacist is practising] *where we don’t really deal with such patients [...] So we deal with it case by case. That’s why […] we never even thought about, to have any…any specific training for that."* (HP06)

#### Domain: Social professional role and identity (SPRI)

Pharmacists had very strong beliefs about dispensing and counselling being their duty and responsibility towards patients, but some had concerns about the understanding of their roles by others, particularly patients/caregivers, physicians, and administrators [Domain: ‘Social influences’]. One pharmacist mentioned the professional boundaries between pharmacists and pharmacy technicians and how the quality of counselling might be compromised when provided by technicians.

*"It is my responsibility that every patient, whether he has a special need or not, does not leave the pharmacy unless he has learned how to use the medication in a correct and effective way."* (HP11)

*“...I don’t want to speak ill of them* [technicians]*, but they don’t have enough information or enough skills to deliver the medicine-related information to the patient."* (HP04)

#### Domain: Beliefs about capabilities

The barriers and facilitators reported under this domain were predominantly linked to availability of environmental resources [Domain: ‘ECR’], having the appropriate knowledge and training [Domains: ‘Knowledge’, ‘Skills’], or factors related to patients/caregivers [Domain: ‘Social influences’].

*"I feel confident when the patient himself has dealt with the drug more than once. So, I dispense it and be rest assured that he will not misuse it, especially if I repeat to him “take it in the morning”..."* (HP09)

*"[...] The more the knowledge you have, I think…and you having received training in a professional manner, I think, the more eager you’d be to deliver what you’ve got to the person in front of you. I think this…I think this is the most influencing factor."* (CP02)

#### Domain: Optimism

Pharmacists’ optimism regarding improvement in dispensing and counselling processes was largely linked to other domains particularly ‘ECR’, ‘Knowledge’ and ‘Skills’. Factors such as the availability of resources and having appropriate education and training were considered facilitators, while workforce shortage, associated costs, and lack of specific guidelines were identified as barriers.

*“That so far, there are no official guidelines for this matter. […] We need to come up with guidelines for this matter, that everybody, like, adheres to." (CP06)*

#### Domain: Beliefs about consequences

All pharmacists were well aware of the consequences of providing appropriate dispensing and counselling services such as improving therapeutic outcomes and patients’ quality of life in addition to enhancing patients’ trust in pharmacists as professionals. However, pharmacists acknowledged that the lack of methods to measure such consequences in practice was a barrier.

*“The quality of his life will increase [...] the costs will decrease [...] the complications will decrease, he will not be readmitted to the hospital.”* (HP01)

*"I cannot know that I made something better if I cannot measure it in the first place."* (HP01)

#### Domain: Reinforcement

Workforce shortage and time constraints, which were noted as barriers under the ‘ECR’ domain were also identified as barriers that discouraged pharmacists from providing appropriate dispensing and counselling services. Maintaining the pharmacy profession reputation, presence of incentives such as appreciation by senior management, and having the appropriate tools to help visually impaired patients were identified as facilitators.

*"...Workforce shortage would not let me sit with every patient and give him his time. So, you’ll find me sitting and trying to finish a number, I mean sitting and finishing umm let’s go, finish the first patient and the next one. Umm, I mean to the point that you’ll sometimes find that there is no counselling and sometimes…and sometimes unfortunately there is no pharmacy [practice]! [...] I mean you’d find me like, sorry for the description, you’d find me like a grocery worker!"* (HP04)

#### Domain: Intentions

It was obvious that no specific services were routinely provided to visually impaired patients across all pharmacy practice settings in Saudi Arabia. Nevertheless, a number of pharmacists described some initiatives to provide such services. Two pharmacists reported attempts at providing label information in Braille at their hospital pharmacies, but both attempts were unsuccessful because of difficulties in obtaining a printer in one case, and the inability to verify the accuracy of printed information in the other. Another initiative involved using QR codes to facilitate access to medication information, but it was yet to be implemented at the time of the interviews. Another pharmacist reported the presence of a specific clinic at the hospital that was equipped with a specialised printer where visually impaired patients could ask for medication instructions to be printed in Braille.

*"One time we started a project which was Braille […] So they brought it so that printing could be in Braille, but the project did not work because of some problems."* (HP11)

#### Domain: Goals

Pharmacists considered providing appropriate dispensing and counselling services to visually impaired patients a priority because they considered such patients to be more prone to medication problems. However, when patients were seen less frequently in the pharmacist’s practice area and in the presence of time constraints, providing appropriate services became less of a priority.

*"Yes! Prio...prio…priority, the highest level of priority. […] Why? Because this is a living example of people who may take medicine in a wrong way, who may have medication errors at a very high rate, at 200% not 100%...soo, high priority."* (HP04)

*"For me, not a very high priority. Because I…I mean, these kind of patients do not come frequently to the pharmacy. That is it. So…this can be improved in hospitals, more of in institutions, I mean.…or the hospital pharmacy, it would of course be better in this regard. Of course they’d consider it a priority. But in a community pharmacy, that would be a little difficult because there are many community pharmacies and these kind of patients may not be that many considering the spread of pharmacies…"* (CP03)

#### Domain: Memory, attention, and decision processes (MADP)

Many factors were identified as facilitators to help pharmacists recognise the needs of visually impaired patients and make appropriate decisions when providing dispensing and counselling services such as: a note about vision impairment in the medical chart, the patient presenting at the pharmacy in person, checking the prescription, and talking with the patient. Factors that prevented pharmacists from appropriately addressing patients’ needs under this domain included: patients not disclosing their vision impairment and pharmacists avoiding asking about it, workload, and focusing on the caregiver rather than the patient.

*"As I am telling you, even when they…when he comes into the pharmacy, he never tells that he is visually impaired [...] and of course I never try to say like [...] “Oh, you have an impaired vision?” …he’d consider it like…an insult or something like that."* (CP04)

*"I give all the directions. But we… for a patient with eye problems, I don’t focus closely because as I am telling you I trust that the caregiver will be the one giving him* [the medication]*."* (HP14)

#### Domain: Environmental context and resources (ECR)

This was the most frequently discussed domain by participants. Some of the barriers and facilitators cited under this domain were also noted in a number of other domains (e.g. ‘Reinforcement’, ‘Goals’, ‘MADP’, ‘Intentions’, ‘Optimism’, ‘Skills’, ‘Knowledge’, ‘Behavioural regulation’). Workload and time constraints were very commonly reported as barriers to medication dispensing and counselling across several domains. Lack of proper tools and resources, access to physicians, the pharmacy layout, and aspects relating to medications (e.g. similar appearance of medications, changing brands) were also considered barriers to provision of appropriate services. Having a guideline or a checklist to guide the dispensing and counselling processes was considered a potential facilitator.

*"Also, providing aids, I mean if there were brochures or video clips or sound, sound clips so that he’d listen to sound clips in one way or other. If there is like…some pharmaceutical accessories like organisers…pill organisers and such..."* (HP04)

*"I would try as much as possible if the packs were similar, two different medications with similar packs, I’d try to look for an alternative with a smaller pack for example…so that they are different in size."* (CP03)

*"...When the pharmacist is providing counselling, there should be like a checklist available to help him with providing the counselling."* (HP04)

#### Domain: Social influences

In the views of pharmacists, caregivers seemed to be important, were frequently referred to in relation to ‘Social influences’ and other domains such as ‘Beliefs about capabilities’ and ‘ECR’. Two pharmacists strongly believed in complete reliance on caregivers for medication management for patients with vision impairment. Patients, caregivers, physicians, and pharmacy colleagues were considered to be both barriers and facilitators depending on their behaviour towards pharmacists. Communication with physicians about prescription issues was an area where differences among pharmacy practice areas were marked. Clinical pharmacists, who typically work in close partnership with physicians in inpatient or outpatient hospital settings [24], reported excellent relationships, demonstrated by physicians ‘relying’ on them in relation to medication issues and accepting suggested changes to medication regimens. Hospital outpatient pharmacists reported easy access to physicians and frequent contact to suggest changes to prescriptions or to clarify information. In contrast, community pharmacists usually reported lack of access to physicians and the absence of means to contact them about prescriptions.

*"I mean there are patients that you feel like they are very careful about themselves, you feel they have the willingness “Yes, tell me. Yes, I want to see…”and some would tell you like “Just finish. I’ll take the medication and leave. It is none of your business. Don’t...” I mean it depends on the attitude of the patient."* (HP03)

*"It is very difficult, impossible to reach the doctor there* [at governmental hospital] *[...] even at private clinics it would be hard to reach him. He is with patients the whole time because of the short operation hours."* (CP04)

*"Basically, even the* [outpatient pharmacy] *supervisor himself wants you to, like, finish patients faster…* ‘*Don’t say…just finish quickly…don’t say…give a lot of information, just give the basics and finish’."* (HP15)

#### Domain: Emotion

Pharmacists reported positive emotions that facilitated their provision of dispensing and counselling services, e.g. feeling satisfied/happy/proud with performing duties, and negative emotions that acted as barriers to provision of appropriate services, e.g. stress which might affect the quality of services and the feeling of increased responsibility which made one pharmacist wish that a supervisor would deal with visually impaired patients.

*"Of course, I feel proud that I, umm was able to contribute to improving his condition, that he would be always safe taking his medicine."* (CP06)

*"I feel like what I’ve been entrusted with has increased. You get it? That…it honestly is a responsibility. I mean you’d wish that these people, [...] like, to have the supervisor to deal with them, maybe especially the blind, or something like that. Because I don’t know, like sometimes you’d wish to relieve yourself of this responsibility. You get it? Sometimes, I mean you are just afraid."* (HP03)

#### Domain: Behavioural regulation

Pharmacists reported a number of methods used to monitor the dispensing and counselling processes such as asking patients to repeat instructions, following-up with patients/caregivers to obtain their feedback, documenting actions, and looking at key performance indicators (KPIs). Pharmacists also highlighted that the lack of formal policies/procedures and methods to measure the effect of counselling were potential barriers.

*"The ability to get the feedback, for example, of the patient or the patient family…that everything is ok and going ok or not? […] Certainly, at the next visit.”* (CP03)

### Identification of key TDF domains to be targeted by an intervention

Based on the data analysis described in Stage 1, all 14 TDF domains were considered relevant to the target behaviour of medication dispensing and counselling. Eight domains were identified as ‘key domains’ to be targeted by a future intervention. These were: ‘Knowledge’, ‘Skills’, ‘Beliefs about capabilities’, ‘Goals’, ‘MADP’, ‘ECR’, ‘Social influences’, and ‘Behavioural regulation’. Table 2 provides a summary of the importance of each of the 14 domains and justification for whether or not they were selected as key domains.

**Table 2:** Importance of TDF domains identified from pharmacists’ interviews

Category	Domains	Comments
Key domains	Knowledge Skills Beliefs about capabilities Goals MADP ECR Social influences Behavioural regulation	● ECR and social influences were the most coded domains ● Beliefs about capabilities: almost all barriers and facilitators under this domain were also linked to other domains particularly ECR, Social influences, Knowledge, Skills
Domains of less importance	SPRI Beliefs about consequences Reinforcement Intentions Emotion Optimism	● SPRI: all pharmacists agreed that dispensing & counselling were part of their job. There was no need for this domain to be targeted further through an intervention ● Beliefs about consequences: all pharmacists were able to cite benefits/risks associated with target behaviours. There was no need for this domain to be targeted further through an intervention ● Reinforcement: It was beyond the capabilities of the project to offer rewards/incentives ● Intentions: all pharmacists had positive intentions. Barriers and facilitators identified under this domain were also identified under other domains ● Emotion: pharmacists expressed positive/negative emotions associated with the target behaviours. It was beyond the scope of the project to target emotions as it would likely require delivery over a long period of time ● Optimism could not be easily targeted in an intervention as it would have required delivery over a long period of time and resources beyond the capabilities of this project. Many barriers and facilitators under this domain were also linked to ECR/social influences

*Abbreviations ECR* Environmental context and resources, *MADP* Memory, attention, and decision processes, *SPRI* Social/professional role and identity

## Discussion

Interviews with pharmacists have highlighted various barriers and facilitators that influence how dispensing and counselling are offered. While the current study findings align with what little has been reported in the literature about factors affecting pharmacists’ services to visually impaired patients [3–5, 13], they are strengthened by following the MRC guidance [9] through utilising the TDF to develop a deep theoretical understanding of the pharmacists’ behaviour. This helped in the identification of previously unreported barriers and facilitators related to issues such as: ‘Beliefs about capabilities’, ‘Reinforcement’, ‘Social influences’ and ‘Emotions’ which were found to influence pharmacists’ medication dispensing and counselling behaviour.

In a 2017 survey of 200 pharmacy staff across the UK, more than two-thirds of the respondents stated that while efforts were made to support visually impaired patients, no routine service was available [2]. The situation in Saudi Arabia seems to be quite similar where no specific services are routinely provided to visually impaired patients across all practice settings.

It was evident that the ‘Knowledge’ and ‘Skills’ domains both needed further enhancement and were identified as key targets for the future intervention. The lack of a consistent method to identify patients with vision impairment in everyday practice may result in overlooking these patients and failure to provide them with appropriate services. These findings are similar to what has been reported in the literature about these patients ‘hiding’ their impairment and not highlighting their needs [4, 30]. Pharmacists in the current study frequently expressed the need for education and training on provision of services to visually impaired patients and that it may need to begin early on during college years. A study published in 2021 by Tang et al*.* [31] found that an educational intervention delivered to Year 2 pharmacy students at the University of Sydney in Australia resulted in significant improvements in eye health knowledge and overall perceptions about pharmacists’ roles in managing people with vision impairment.

Patients’ resistance to acknowledge pharmacists as an important part of the healthcare team and their beliefs that physicians are more knowledgeable and well-trained than pharmacists have been reported to influence patients’ perceptions [32]. Moreover, negative public perception towards pharmacists in Middle Eastern countries was noted in a number of studies included in a systematic review by El Hajj et al*.* [33] where participants had a low level of trust in information provided by pharmacists. Limited knowledge of pharmacists’ training by other professionals has been cited as one of the challenges of pharmacist-physician collaborations [34] and pharmacists have been encouraged to clarify their own clinical roles, establish trustworthiness, and seek positive interactions in order to develop connections with other clinicians [35].

As previously stated, ‘ECR’ was the most commonly discussed domain by participants. Features of the pharmacy layout noted as barriers under ‘ECR’, e.g. lack of privacy and noise levels have also been reported by Dagnachew et al*.* [13] in interviews with community pharmacists in Ethiopia and by Alhusein et al*.* [4] in interviews with community pharmacy personnel in Scotland. The frequent reference to workload and time constraints as barriers to medication dispensing and counselling across several domains is again consistent with the findings of Alhusein et al*.* [4], who identified these factors as barriers to provision of pharmaceutical services to people with sensory impairment and with other TDF-based studies focusing on pharmacists’ behaviour [25, 26]. It is important to consider the time and amount of work required for a future intervention for it to be acceptable to pharmacists and easily embedded within everyday practice.

The importance of caregivers' involvement was evident throughout the interviews and reflected the findings of Lee and Lee [5] who indicated that approximately 60% of pharmacists provided counselling to the patient’s family or caretakers and attributed that to two possible issues: lack of basic understating of vision impairment and failure to recognise it in patients. The latter was also reflected under the domain of ‘Knowledge’ in the findings.

All 14 TDF domains were considered relevant since they were often coded during transcript analysis and the identified barriers and facilitators were thought to have a strong impact on how pharmacists dispense and counsel medications for visually impaired patients. The eight domains identified as ‘key domains’ were perceived as feasible targets for a future intervention within the proposed intervention setting (outpatient hospital pharmacies or community pharmacies) and the resources that were available. Six TDF domains were deemed less important to target in a future intervention for a number of reasons. For example, all pharmacists identified many positive consequences for appropriate dispensing and counselling and negative consequences associated with inappropriate dispensing and counselling. Hence, we did not feel that the ‘Beliefs about consequences’ domain needed to be further addressed through a future intervention. When it came to ‘Reinforcement’, no resources were available to offer monetary incentives. Offering continuing education hours (CMEs) to pharmacists was considered as an alternative because it was suggested by one of the participants during interviews. However, the process of assigning CMEs to educational activities in Saudi Arabia is a somewhat complicated and lengthy process that would have significantly affected the study timeframe. Therefore, it was decided not to include ‘Reinforcement’ as one of the key domains. Targeting ‘Emotions’ would have also affected the timeline because changes in emotion require long periods of time to occur.

### Strengths and limitations

To the authors’ knowledge, this is the first study to utilise theoretical underpinning to explore pharmacists’ behaviours in relation to medication dispensing and counselling for patients with vision impairment. However, the qualitative nature of the study affects the transferability of the findings to the international pharmacist community. The study followed the systematic approach recommended by the earlier version of the UK’s MRC framework for developing interventions to improve health [9]. The guidance was updated in 2021 after this study had concluded [21]. Female pharmacists were under-represented in this study, constituting around 31% of the total sample. All nine community pharmacists in the sample were male. This reflects the fact that the number of males in the Saudi pharmacy workforce is more than four times higher than the number of females [36]. Moreover, female pharmacists were only recently allowed to work in community pharmacy practice settings [37]. The study included pharmacists from different practice areas and settings (e.g. hospital vs. community; outpatient hospital pharmacy vs. clinical pharmacy). While pharmacists from private hospital settings were not interviewed, it should be noted that the majority of outpatient pharmacists involved with dispensing and counselling within that setting, practise in what essentially is a community pharmacy located on or near the premises of a private hospital. Depending on their practice setting, pharmacists with limited experience may have had relatively little interaction with patients who have vision impairment.

Two types of triangulation were conducted: investigator triangulation (by having a number of transcripts independently double coded by two members of the research team), and environmental triangulation (by interviewing pharmacists from different practice settings and practice areas). This addressed the credibility and confirmability of the findings. To enhance transferability and authenticity, a ‘thick description’ was provided by detailing the different settings from which pharmacists were recruited, ensuring that quotes reflected the various facilitators/barriers expressed by pharmacists, and describing the reasoning underpinning the decisions that were made. The dependability and authenticity of the findings were increased by maintaining an audit trail of all data generated throughout the study such as audio recordings, transcripts, translations, and analysis, and by decisions being reached through consensus. Because recruitment and interviewing continued over a long period of time, it was not possible to carry out ‘member checking’, i.e. to ask participants to review transcripts or comment on the final results.

## Conclusions

This study adopted a qualitative approach to develop an in-depth understanding of pharmacists’ dispensing and counselling behaviour for patients with vision impairment. Utilising the TDF as the theoretical lens, a number of barriers and facilitators have been identified, with eight domains considered important to target in a future intervention. Further research will focus on mapping key TDF domains identified through this study to BCTs using the mapping matrix developed by Michie et al*.* [17] and Cane et al*.* [20]. These BCTs which will then be incorporated into a proposed intervention to support the processes of medication dispensing and counselling to visually impaired patients.

### Supplementary Information

**Additional file 1.** COREQ checklist. The 32-item Consolidated criteria for reporting qualitative research.

**Additional file 2.** Pharmacists’ interview topic guide. TDF-based topic guide used during pharmacists’ interviews to explore barriers and facilitators to medication dispensing and counselling.

**Additional file 3.** Expanded version of the barriers and facilitators identified under each of 14 TDF domains. A table with additional barriers and facilitators under each of the TDF domains together with illustrative quotes.

## Data Availability

The data that supports the findings of this study are available on reasonable request from the corresponding author. The data are not publicly available due to them containing information that could compromise research participant privacy/consent.

## Abbreviations

CMEs: Continuing education hours
COREQ: Consolidated criteria for reporting qualitative research
CP: Community pharmacist
ECR: Environmental Context and Resources
HP: Hospital pharmacist
MADP: Memory, attention, and decision processes
MOH: Ministry of Health
MRC: Medical Research Council
SPS: Saudi Pharmaceutical Society
SPRI: Social/professional role and identity
TDF: Theoretical Domains Framework
UK: United Kingdom
