# Supplementary material for: Improving medication dispensing and counselling for patients with vision impairment: a qualitative study of pharmacist-reported barriers and facilitators
Source: BMC Health Serv Res. 2024 Apr 26;24:534. doi: 10.1186/s12913-024-11009-9 (PMC11046806; doi:10.1186/s12913-024-11009-9)
Supplement: Supplementary file 1 — Additional file 1. COREQ checklist. The 32-item Consolidated criteria for reporting qualitative research. [file 12913_2024_11009_MOESM1_ESM.pdf]

Additional File 1: COREQ checklist

No. Item	Guide questions/description	Comments
**Domain 1: Research team and reflexivity**		
Personal Characteristics		
**1. Inter viewer/facilitator**	Which author/s conducted the interview or focus group?	One researcher (BK) conducted all interviews.
**2. Credentials**	What were the researcher's credentials? e.g. PhD, MD	BK had a BSc. in Pharmaceutical Sciences and MSc. in Clinical Pharmacy and was a PhD student at the time of the study.
**3. Occupation**	What was their occupation at the time of the study?	BK was a lecturer at the College of Pharmacy, King Saud University and a PhD student at Queen's University Belafast.
**4. Gender**	Was the researcher male or female?	Female.
**5. Experience and training**	What experience or training did the researcher have?	BK had undertaken training in qualitative research methodologies.
Relationship with participants		
**6. Relationship established**	Was a relationship established prior to study commencement?	No prior relationship was established prior to the contact about the study.
**7. Participant knowledge of the interviewer**	What did the participants know about the researcher? e.g. personal goals, reasons for doing the research	Participants were aware that BK was a lecturer and a PhD student and were briefed on the purpose of the study.
**8. Interviewer characteristics**	What characteristics were reported about the interviewer/facilitator? e.g. Bias, assumptions, reasons and interests in the research topic	BK had an interest in the research topic.
**Domain 2: Study design**		
Theoretical framework		
**9. Methodological orientation and Theory**	What methodological orientation was stated to underpin the study? e.g. grounded theory, discourse analysis, ethnography, phenomenology, content analysis	The Theoretical Domains Framework underpinned the development of the study. Framework analysis was carried out followed by content analysis.
Participant selection		
**10. Sampling**	How were participants selected? e.g. purposive, convenience, consecutive, snowball	Purposive and snowball sampling were utilised.
**11. Method of approach**	How were participants approached? e.g. face-to-face, telephone, mail, email	Participants were contacted via telephone or email followed by a formal invitation letter through email or WhatsApp messages.
**12. Sample size**	How many participants were in the study?	26 pharmacists
**13. Non-participation**	How many people refused to participate or dropped out? Reasons?	All pharmacist who were approached after meeting the inclusion criteria completed the study.

No. Item	Guide questions/description	Comments
Setting		
**14. Setting of data collection**	Where was the data collected? e.g. home, clinic, workplace	Pharmacists were interviewed at their place of work or over the telephone.
**15. Presence of non-participants**	Was anyone else present besides the participants and researchers?	No
**16. Description of sample**	What are the important characteristics of the sample? e.g. demographic data, date	Participants were pharmacists from different pharmacy practice areas (community, hospital, clinical, managerial). Eighteen were males and eight were females.
Data collection		
**17. Interview guide**	Were questions, prompts, guides provided by the authors? Was it pilot tested?	A topic guide with prompts was developed and used during the semi-structured interviews. The topic guide was piloted with three pharmacists.
**18. Repeat interviews**	Were repeat inter views carried out? If yes, how many?	No repeat interviews were required.
**19. Audio/visual recording**	Did the research use audio or visual recording to collect the data?	Interviews were audio-recorded.
**20. Field notes**	Were field notes made during and/or after the inter view or focus group?	BK recorded field notes during and after the interviews.
**21. Duration**	What was the duration of the inter views or focus group?	Interviews' duration ranged from 39 minutes to two hours and 20 minutes.
**22. Data saturation**	Was data saturation discussed?	Data saturation was achieved at interview number 26.
**23. Transcripts returned**	Were transcripts returned to participants for comment and/or correction?	Transcripts were not returned to participants.
**Domain 3: Analysis and findings**		
Data analysis		
**24. Number of data coders**	How many data coders coded the data?	Two researchers (BK and CH or HB) coded three interviews initially and then BK coded the remainder of the interviews.
**25. Description of the coding tree**	Did authors provide a description of the coding tree?	Data was reported under the TDF domains and barriers/ facilitators were identified under each domain.
**26. Derivation of themes**	Were themes identified in advance or derived from the data?	TDF domains represented the main themes under which barrier/facilitators were identified from the data.
**27. Software**	What software, if applicable, was used to manage the data?	NVivo® QSR 12
**28. Participant checking**	Did participants provide feedback on the findings?	Future publications will be made accessible to participants.
Reporting		
**29. Quotations presented**	Were participant quotations presented to illustrate the themes/findings? Was each quotation identified? e.g. participant number	Quotations were presented within the results section with participants given an anonymous code (e.g. CP04, HP13).
**30. Data and findings consistent**	Was there consistency between the data presented and the findings?	See results and discussion sections. Efforts were taken to present data in a clear and consistent manner highlighting where differences were identified.
**31. Clarity of major themes**	Were major themes clearly presented in the findings?	
**32. Clarity of minor themes**	Is there a description of diverse cases or discussion of minor themes?	
