# Supplementary material for: Improving medication dispensing and counselling for patients with vision impairment: a qualitative study of pharmacist-reported barriers and facilitators
Source: BMC Health Serv Res. 2024 Apr 26;24:534. doi: 10.1186/s12913-024-11009-9 (PMC11046806; doi:10.1186/s12913-024-11009-9)
Supplement: Supplementary file 2 — Additional file 2. Pharmacists’ interview topic guide. TDF-based topic guide used during pharmacists’ interviews to explore barriers and facilitators to medication dispensing and counselling. [file 12913_2024_11009_MOESM2_ESM.pdf]

Additional File 2: Pharmacists' interview topic guide

Introduction

My name is Basma Kentab. I am a lecturer at the College of Pharmacy, King Saud University and I am conducting this study as part of my PhD studies at Queen's University Belfast, UK. Thank you for taking the time to speak with me today.

In this study we are speaking to patients with visual impairment and their caregivers to explore how patients get and use their medicines. For the purposes of this study, we describe the caregiver as someone who has contact with the patient with sight problems at least three times a week and provides help to the patient with their medicines. We will also speak to pharmacists to find out about their experiences of dispensing and counseling patients with visual impairment or their caregivers. We plan to use the information gathered during this study to come up with ways to help these patients get the most out of their medicines.

Have you had a chance to read through the information sheet that was sent to you? Are there any questions that you would like to ask me?

Explaining what will happen in the interview and afterwards

The aim of this interview is to explore your views of medicines optimisation in patients with visual impairment, your current practice of dispensing and counseling to this patient population, and your perspectives on what might help or prevent medicines optimisation for patients with visual impairment. The definitions of both visual impairment and medicines optimisation will be provided to you before starting the interview to help explain what they mean in the context of this study. The interview should last about 45 minutes.

I will be recording the interview on a digital recorder, to ensure that we have an accurate record of what is discussed. The recording will be saved on a password-protected computer and only those directly involved in the research study will listen to it. The recording will be typed up word-for-word and any names, locations, or anything else that could identify you or anyone you talk about will be removed. Your participation is voluntary, and you are free to stop the interview at any time or skip any question you don't wish to answer. After we complete the interviews of all participants, we will analyse the information and results may be published in scientific journals and conferences.

I have here your written consent indicating that you agree to participate in the study and have this interview audio-recorded. [check that the written consent form is appropriately signed and dated]

Do you have any questions or would like any additional details before we start? [Answer questions.]

[Turn the digital recorder on]

I will start by giving you some relevant definitions to help you have the appropriate context for the questions that follows.

Definitions

<u>Visual impairment:</u> There is no universally accepted definition of visual impairment, but for the purposes of this study we are using the World Health Organization 2016 classification where we are interested in visual impairment defined as visual acuity of < 6/18 or equivalent, measured with glasses or contact lenses if they are used by the patient. A score of 6/18 means that the person can see at 6 meters what a person with normal vision can see from 18 meters. This definition will include people with moderate visual impairment, severe visual impairment or blindness.

<u>Medicines optimisation:</u> This is defined by the British National Institute for Health and Care Excellence (NICE) as 'a person-centred approach to safe and effective medicines use, to ensure people obtain the best possible outcomes from their medicines'. The goal of medicines optimisation is to help patients to: 'improve outcomes related to medicine use; take their medicines correctly; avoid taking unnecessary medicines; reduce wastage of medicines; and improve medicines safety'.

Demographic Information

- Gender (To be recorded on separate note for each participant)
- Can you tell me how long have you been practicing as a pharmacist?
- What type of pharmacy setting are you currently working at?
  - *Prompt:* Community/hospital/primary healthcare centre, governmental/private?
- Where is your pharmacy located?
  - *Prompt:* Region, city, neighbourhood?
- Do you know if any patients with visual impairment visit your pharmacy?
- Have you completed any additional training (either formal i.e. lectures or workshops or self-learning) in the care of patients with visual impairment?

Behavioral elicitation

"Before we begin, it would be useful if you could think about a situation where you have dispensed medicines and provided counseling to a patient who is visually impaired. You may have also dealt with the patient caregiver. If you can't think of a specific situation, don't worry, just think about medicines optimisation for patients with visual impairment in general terms using the definitions I've given you"

Knowledge

- How would you know whether a patient has visual impairment?
- What do you think are the challenges or risks that a patient with visual impairment faces when they are prescribed medicines?
- What knowledge do you think you require as a pharmacist that would help you when dispensing medicines and counseling a patient with visual impairment?
    - *Prompts: clinical knowledge (i.e. knowledge of visual impairment and how it affects patient health and medicines)*
        - Specific knowledge sources/resources
        - Is there anything specifically relating to patients with visual impairment?
    - *Prompt*: knowledge of the extent of the patient's visual impairment.
    - *Prompts*: knowledge of guidelines (specific to visually impaired patients)
        - What guidelines?
    - *Prompt*: Personal knowledge/experience of visual impairment.
        - What effect does this have on your practice?

Skills

- What skills do you have as a pharmacist to assist you when dispensing medicines for and counseling a patient with visual impairment and dealing with any issues that may arise with dispensing or counseling?
    - *Prompt*s
        - What skills do you have that would help you to interact with patients or their caregivers?
        - What skills do you have that would help you to interact with physicians?
- Is there any specific training or support that you feel would be helpful to you in order to improve dispensing and counseling for patients with visual impairment in the future?

Social/professional role and identity

- Thinking about medicines optimisation for patients with visual impairment, what would you consider your responsibilities to be as a pharmacist in ensuring that patients with visual impairment and their caregivers receive appropriate dispensing and counselling for their medicines?
    - *Prompt*
        - Is there anything that you would consider to be beyond (or outside) your responsibility as a pharmacist (in ensuring that patients with visual

impairment and their caregivers receive appropriate dispensing and counselling for their medicines)?

        - Who do you think is responsible for these aspects beyond your responsibility?

Beliefs about capabilities

- In what situations would you feel confident about dispensing medicines and counselling a patient with visual impairment?
- In what situations would you NOT feel confident about dispensing medicines and counselling a patient with visual impairment?
    - *Prompt*
        - What would help you overcome these problems or difficulties?

Optimism

- How optimistic (or hopeful) are you that appropriate dispensing and counselling can be achieved for patients with visual impairment?
- What would make you feel less optimistic (or hopeful) that appropriate dispensing and counselling can be achieved for patients with visual impairment?

Beliefs about consequences

- What do you think are the benefits of appropriate dispensing and counselling for patients with visual impairment?
    - *Prompt:* For patients; their caregivers; yourself; short- and long-term consequences
- What are the risks associated with inappropriate dispensing and counselling for patients with visual impairment?

Reinforcement

- What would encourage you to ensure dispensing and counselling are appropriate for patients with visual impairment?
    - *Prompt:* Are there any rewards or incentives for you or the pharmacy, e.g. additional income, personal rewards, professional recognition by your supervisor?
- What would discourage you from ensuring dispensing and counselling are appropriate for patients with visual impairment?

Intentions

- How would you plan to address issues with dispensing and counselling for patients with visual impairment?
- What would prevent you from addressing issues with dispensing and counselling for patients with visual impairment?

Goals

- To what extent is improving dispensing and counselling for patients with visual impairment a priority for you?
  - *Prompt:*
    - If low/high priority, why?
    - Are improving dispensing and counselling in this patient group more important than any other patient group?
- In what circumstances would you think it was less important to make any changes to dispensing and counselling for patients with visual impairment?

Memory, attention and decision processes

- How would you usually remember to address issues with dispensing and counselling for patients with visual impairment?
  - *Prompt:* For example, computer alerts, notes or memos
- Are there any circumstances in which you might find it difficult to remember to resolve these issues?

Environmental context and resources

- What resources or support might help you when you encounter issues with dispensing and counselling in patients with visual impairment?
  - *Prompt:* For example, medicines labels and leaflets in large font or in Braille, labels and leaflets in audible format, different coloured tape or rubber bands to place on medicines vials.
- What factors might prevent you from intervening when you encounter issues with dispensing and counselling in patients with visual impairment?
  - *Prompt:* Work environment, material resources not available, time issues

Social influences

- Who would influence your decisions about dealing with issues with dispensing and counselling for patients with visual impairment?
  - *Prompt:* Patients; caregivers or relatives; physician; pharmacy colleagues; other healthcare professionals
  - *Prompt:* Can you tell me more about how this happens and what their influence is?

Emotion

- How does dispensing and counselling for patients with visual impairment make you feel?
  - *Prompt:* worried, stressed, satisfied with doing your job

- How would your own work stress or emotional engagement with a patient and their caregiver influence your decisions to attempt to resolve issues with dispensing and counselling for patients with visual impairment?

Behavioural regulation

- Having decided the best course of action to resolve dispensing and counselling issues for a patient with visual impairment, are there any ways in which you can monitor whether or not it has been done?
    - ***Prompt*:** Following clinical guidelines or workplace protocols

Intervention components

"The research team is interested in developing an intervention (or plan) *to support and improve medicines optimisation for patients with visual impairment and their caregivers. From reviewing the literature, we have found that, generally, interventions can be complex and often involve a number of different components. This makes it difficult to identify which components are the most important in terms of improving patient outcomes and achieving adequate medicines optimisation."*

- What would you consider to be important components of an intervention (or plan) to support medicines optimisation (particularly dispensing and counselling) for patients with visual impairment and their caregivers?
    - *Prompt:* Who should be involved in delivering these types of interventions (or plans) in practice (caregivers, pharmacists, physicians, nurses, charities)?
    - *Prompt:* What would each person/healthcare professional have to do?
    - *Prompt:* What are your thoughts on patient involvement in interventions (or plans) – should patients with visual impairment be actively involved in the decisions about the medicines they are using?
- What factors would help putting the type of intervention (or plan) that you have described into practice?
- What factors would prevent putting the type of intervention (or plan) that you have described into practice?
- What do you think should be measured as an outcome in an intervention study to support medicines optimisation for patients with visual impairment, i.e. how would you, personally, be persuaded that the intervention (or plan) had made a difference to medicines optimisation (particularly dispensing and counselling)? What are the important outcomes?

Closing the interview

"That brings us to the end of the interview.

Is there anything else about medicines optimisation in people with visual impairment that you feel has not been covered?

Do you have any additional comments you would like to make as to the content of the interview or how it went?

Thank you very much for making the time to speak with me today." [Turn the digital recorder off]
