# Supplementary material for: Improving medication dispensing and counselling for patients with vision impairment: a qualitative study of pharmacist-reported barriers and facilitators
Source: BMC Health Serv Res. 2024 Apr 26;24:534. doi: 10.1186/s12913-024-11009-9 (PMC11046806; doi:10.1186/s12913-024-11009-9)
Supplement: Supplementary file 3 — Additional file 3. Expanded version of the barriers and facilitators identified under each of 14 TDF domains. A table with additional barriers and facilitators under each of the TDF domains together with illustrative quotes. [file 12913_2024_11009_MOESM3_ESM.pdf]

## Additional File 3: Expanded version of the barriers/facilitators identified under each of 14 TDF domains

### Determinants (facilitators and barriers) of pharmacists' medication dispensing and counselling behaviour

| TDF Domain | Facilitators | Barriers | Illustrative quotes |
|---|---|---|---|
| **Knowledge** | Knowledge of presence of VI in a patient (patient behaviour, medical chart/prescription, appearance, caregiver presence, other HCPs, diagnosis/comorbidities, disability card)<br><br>Knowledge of level of VI<br><br>Knowledge of challenges/risks to patients<br><br>Knowledge of home support available to patient/patient situation<br><br>Clinical knowledge, knowledge of medications and patient history<br><br>Knowledge of guidelines<br><br>Knowledge of patient preferences/needs<br><br>Experience | Barriers to knowing that patient has VI (female patients covering face, patients not disclosing their VI, pharmacist not asking about VI to avoid embarrassing patients, VI patients not picking up medicines themselves, no alerts on computer system)<br><br>Lack of guidelines, no knowledge of guidelines<br><br>Lack of access to patient data<br><br>Lack of education about the issue in pharmacy colleges | *"Umm I mean this is the most important thing, to know that he has visual impairment."* (CP06)<br><br>*"No, no one mentions something like that. I don't think that he even likes disclosing it to someone. [...] So I feel they don't tell out of embarrassment, so I avoid stating it. I mean I deal with him based on what I see in front of me and that is it…or what I felt from him."* (CP04)<br><br>*"It would be written in the notes, but, [...] nobody will access the doctor's notes and read it to know if this information is present or not. And it is not like an allergy, it is not included under allergies or the complications present in the…in the head* [of the page]*."* (HP05)<br><br>*"Yes, it* [level of impairment] *is very important so… because it will change the kind of patient counselling."* (HP08)<br><br>*"I have to know the challenges they have…then I can [...] decide"* (HP06)<br><br>*"My exp…clinical experience, ok, as clinical pharmacist…this is…ok, understanding the diseases, understanding, ok, the need of my colleagues…umm and also reading the cases…reading the case of the patient''* (HP06)<br><br>*"I mean, it would be easier for me if there were a guideline."* (HP05) |

| Determinants (facilitators and barriers) of pharmacists' medication dispensing and counselling behaviour | | | |
|---|---|---|---|
| **TDF Domain** | **Facilitators** | **Barriers** | **Illustrative quotes** |
| **Skills** | Patience, tolerance, gaining patient trust and quick-wittedness<br><br>Communication skills (with patients/physicians)<br><br>Interpersonal skills<br><br>Counselling skills (e.g. listening, giving more time, simplifying language, describing shapes of tablets, have patient touch medicines)<br><br>Problem-solving skills<br><br>Future courses/training to improve skills<br><br>Experience in practice | Lack of training | *"I think communication is the most important thing. That you should not be, like, annoyed with the person in front of you, or appreciate his condition. So, you should be a little patient."* (CP04)<br><br>*"Yeah, the most important thing is that my counselling is, like, slow and clear to the patient. And if he does not understand, that is fine, I'd repeat things for him again, even if I took longer with him, I don't have a problem."* (HP13)<br><br>*"If I were […] trained or had read a more professional guideline for example um and had gotten a background about how to deal with this patient in a better way. Because the ways I have…are like ways…like they deliver the point but they are not professional to the point that…yeah."* (HP03)<br><br>*"…if you are in an institution umm where you probably see many patients, […] then in this case umm, ok, …there should be training, there should be… using all tools that might…that might help. But for…let's say at* [name of hospital where pharmacist is practicing] *where we don't really deal with such patients [...] So we deal with it case by case. That's why […] we never even thought about, to have any…any specific training for that."* (HP06) |
| **SPRI** | Professional responsibility/duty for dispensing/counselling and all activities involved<br><br>Moral/religious beliefs in relation to role<br><br>Maintaining pharmacy profession reputation | Senior management not understanding pharmacist role (hospital pharmacy)<br><br>Interprofessional relationship with physicians | *"It is my responsibility that every patient, whether he has a special need or not, does not leave the pharmacy unless he has learned how to use the medication in a correct and effective way."* (HP11)<br><br>*"God gave you this task to help those people, so you need to be up to this confidence that God has entrusted in you."* (CP02)<br><br>*"But you get worried about the profession as a whole, and about the image of the profession too. And we want this to never shake. People's confidence in the pharmacy, we don't want that to be shaken no matter what."* (HP16) |

| Determinants (facilitators and barriers) of pharmacists' medication dispensing and counselling behaviour | | | |
|---|---|---|---|
| **TDF Domain** | **Facilitators** | **Barriers** | **Illustrative quotes** |
| **SPRI (*cont'd*)** | Roles of others (e.g. technicians for preparing medicines, physicians for consultation)<br><br>Beliefs that pharmacists are the most suited to counsel patients | Professional boundaries between pharmacists and technicians (beliefs that when technicians provide counselling, quality is compromised)<br><br>Lack of understanding of pharmacist's role by patients/caregivers<br><br>Pharmacists are not currently required to provide special treatment for VI patients | *"I would go back and talk to the…the resident, the intern, anyone…whoever made the prescription, I'd go back and talk to him to be able to change or alter the dose, to completely change the medication… [...] because I am with them in the team. So, when I tell him "Well, because there is an alternative", he'd choose that…he takes my word for it, I mean"* (HP05)<br><br>*"...One of the main problems is the lack of understanding of the higher administration or other entities of the pharmacist's roles [...] I mean, they do not realise the importance of your role as a pharmacist."* (HP04)<br><br>*"...So, we can…alert him to the medication error present in the prescription. There is a sort of reluctance to accept this, ''I am a physician, and you are a pharmacist!''. I mean he does not realise what the pharmacist* [role] *is. He does not realise that medical information and disease are his speciality and medicine-related information is the pharmacist's speciality. And this happens alooooot* [sic]*, at a very high rate!"* (HP04)<br><br>*"…I don't want to speak ill of them* [technicians]*, but they don't have enough information or enough skills to deliver the medicine-related information to the patient. "* (HP04)<br><br>*"[...] He* [patient] *insists on using it or tells me "I want it, none of your business!" or that the physician also tells me "Nope, dispense what I have written"…that is something considered… that is their wishes. In that case, I cannot…I mean I wouldn't… and in spite of that I'll continue to offer advice".* (CP02) |
| **Beliefs about capabilities** | Patient understanding, patient satisfaction<br><br>Patient prior use of medication<br><br>Caregiver/family caring for patient | Not having enough information about medication<br><br>Unclear prescription | *"So, he* [patient] *explains everything back to me while I'm there. So, some would explain…when I see him hesitating or something, no, here there was a problem with my work. Like I'd be confident, and it makes my day, the one who races me to say the information"* (HP16)<br><br>*"Look, I would never be confident, because he cannot see. But, I would be more reassured if one of his family was around, if he audio recorded me, if he recorded a video…"* (HP05) |

| Determinants (facilitators and barriers) of pharmacists' medication dispensing and counselling behaviour | | | |
|---|---|---|---|
| **TDF Domain** | **Facilitators** | **Barriers** | **Illustrative quotes** |
| **Beliefs about capabilities (*cont'd*)** | Having the right resources/tools<br><br>Having the necessary knowledge/training<br><br>Sufficient and clear information about the patient's case | Instances requiring a referral to a physician, complicated cases<br><br>Caregiver (caregiver absent or may misunderstand information)<br><br>Feeling of increased responsibility<br><br>No previous experience with VI patients<br><br>Patient being completely blind or having multiple disabilities | *"I feel confident when the patient himself has dealt with the drug more than once. So I dispense it and be rest assured that he will not misuse it, especially if I repeat to him "take it in the morning"..."* (HP09)<br><br>*"[...] The more the knowledge you have, I think...and you having received training in a professional manner, I think, the more eager you'd be to deliver what you've got to the person in front of you. I think this...I think this is the most influencing factor".* (CP02)<br><br>*"If it is something that is outside our scope as pharmacists, I mean. For example, he is complaining of certain symptoms ... umm and these require a specialist"* (CP01)<br><br>*"If that other person that is with them is like...suppose he was driver of a different nationality, umm cannot understand Arabic well or his English was weak, surely he would not understand the information correctly!"* (CP03)<br><br>*"I feel like what I've been entrusted with has increased. You get it? That...it honestly is a responsibility. I mean you'd wish that these people, [...] like to have the supervisor to deal with them, maybe especially the blind, or something like that. Because I don't know, like sometimes you'd wish to relieve yourself of this responsibility. You get it? Sometimes, I mean you are just afraid."* (HP03) |
| **Optimism** | Availability of resources (e.g. technologies and use of Braille)<br><br>Education/training<br><br>Complete circle of care (physician, pharmacist, patient, caregiver)<br><br>Continuous development in healthcare services | Long standing issue<br><br>Cost<br><br>Workforce shortage<br><br>Pharmacist having other duties<br><br>Lack of guidelines | *"It can* [be] *improved, but you need...you need manpower and you need budget! (laughs) I mean ideas remain ideas unless I mean you have actions."* (HP10)<br><br>*"Umm when the ...umm he* [patient] *comes out of the physician's* [clinic] *with a prescription that is written well...clear font. When the pharmacist also has enough information about how to deal with the visually impaired. Umm when the patient himself knows how to take the medicine, has a caregiver or something or he himself knows how to take the medicine. Umm I think that... if this circle is complete, I mean it would be good."* (CP06) |

| Determinants (facilitators and barriers) of pharmacists' medication dispensing and counselling behaviour | | | |
|---|---|---|---|
| **TDF Domain** | **Facilitators** | **Barriers** | **Illustrative quotes** |
| **Optimism (*cont'd*)** | Community/organisation interest in helping these patients | Lack of support (e.g. understanding from senior management, charities support), bureaucracy<br><br>Lack of training/education about issue in pharmacy colleges<br><br>Pharmacists' behaviour (e.g. disinterest) | *"I mean all matters are evolving for the better, especially that there are decisions that this category should have its right in society. Thus…I mean…I think the companies from now on will have all instructions written on the medicine itself"* (HP09)<br><br>*"Well, 50%. […] Because the workload is high. […] In terms of the number of patients, in terms of the pharmacist not being freed just for counselling. He has other things [to do]. So, he just barely gives the main points and then carries on with his work. You get it?"* (HP03)<br><br>*"The interest! [...] Like we did not hear about courses or colleges or something offering education about this. [...] I mean the community role is also non-existent."* (CP05)<br><br>*"That so far, there are no official guidelines for this matter. I think similar to guidelines made for using any medicine, there should be one for patients…with visual impairment. We need to come up with guidelines for this matter, that everybody, like, adheres to."* (CP06) |
| **Beliefs about consequences** | Patient taking medication appropriately<br><br>Improved management of conditions and quality of life<br><br>Reduced costs/complications<br><br>Improved therapeutic outcomes for patients<br><br>Improved patient compliance<br><br>Patient's trust in pharmacist | Lack of methods to measure consequences | *"Umm without counselling, I do not think he will take his medication correctly."* (HP15)<br><br>*"The quality of his life will increase [...] the costs will decrease [...] the complications will decrease, he will not be readmitted to the hospital"* (HP01)<br><br>*"It will relief him* [caregiver] *psychologically, ok. It will save his time, ok. I mean it will save his effort and the service he provides to the person he is caring for."* (CP05)<br><br>*"Secondly, on the pharmacist, the pharmacist would lose trust…the patient would lose his trust in him so… and it would extend to pharmacists in general not only this pharmacist. So, he* [patient] *would believe for example that "Nope, the pharmacist did not offer me the right service or so on" so he would lose confidence in pharmacists generally as professionals."* (CP06) |

| Determinants (facilitators and barriers) of pharmacists' medication dispensing and counselling behaviour | | | |
|---|---|---|---|
| **TDF Domain** | **Facilitators** | **Barriers** | **Illustrative quotes** |
| **Beliefs about consequences (*cont'd*)** | Reduced caregiver burden<br><br>Enhanced pharmacist's satisfaction<br><br>Reduced pharmacist's liability<br><br>Improving pharmacist's skills and experience<br><br>Rewards from God for good deeds/clear conscience | | *"Um like you would be experienced with this, and honestly feel self-satisfied and that you have achieved something."* (HP15)<br><br>*"A problem might happen to him as a result. [...] It would be said that I am the cause of this problem. That would be a personal problem first. Of course, as a general problem um it might even affect the pharmacy group I work for."* (CP03)<br><br>*"And from…from our believes* [sic] *that we can get rewards or…get rewards from your Allah if we…you do good deeds."* (HP08)<br><br>*"I cannot know that I made something better If I cannot measure it in the first place."* (HP01) |
| **Reinforcement** | Professional responsibility<br><br>Maintaining pharmacy profession reputation<br><br>Patient improvement/ satisfaction/trust<br><br>Incentives (depending on person's preference) e.g. employee of the month, verbal encouragement<br><br>Having the tools to help patients<br><br>Personal rewards from God for doing good, justify salary<br><br>Computer system reminders | Workforce shortage<br><br>Time constraints/lack of privacy<br><br>No appreciation of pharmacist efforts<br><br>Lack of patient/family knowledge about medicines<br><br>Patient's behaviour<br><br>Amount of effort required to deal with VI patients<br><br>Administration caring more about numbers served, meeting KPI targets | *"...The pharmacist is supposed to assert or ensure that the patient has received the information correctly, apart from…apart from incentives and such, professionally! I mean it's one of the principles of the profession and one of the duties of the profession, that he needs to assert and ensure that the patient has received the information correctly."* (HP04)<br><br>*"…Like, improvement of the profession…that the pharmacist is capable of, like, … providing counselling for all categories of the society"* (HP03)<br><br>*"If I have the tools…If I have the tools, yeah to help them."* (HP06)<br><br>*"No, this is not present. But if it were present, it would be nice. […] Depends on the recipient, on the employee himself. […] I mean some would say I want the money and others would say no, well, an appreciation letter, like, would be good."* (HP04)<br><br>*"What is discouraging is that, well, whether I work or not, like, I'm treated the same as anyone else. Nobody is seeing what I…how much I'm providing to the patient."* (HP11) |

| Determinants (facilitators and barriers) of pharmacists' medication dispensing and counselling behaviour | | | |
|---|---|---|---|
| **TDF Domain** | **Facilitators** | **Barriers** | **Illustrative quotes** |
| **Reinforcement (*cont'd*)** | | Comments from colleagues | *"...Workforce shortage would not let me sit with every patient and give him his time. So, you'll find me sitting and trying to finish a number, I mean sitting and finishing umm let's go, finish the first patient and the next one. Umm I mean to the point that you'll sometimes find that there is no counselling and sometimes…and sometimes unfortunately there is no pharmacy [practice]! [...] I mean you'd find me like, sorry for the description, you'd find me like a grocery worker!"* (HP04)<br><br>*"For us here, timing is usually important. Because…because we have the K…the KPI which must be like less than 20 minutes [...] So I think like this is a little not ummm is against the pharmacist doing counselling with every patient."* (HP17) |
| **Intentions** | Positive intentions to resolve issues related to dispensing & counselling | Time pressure<br><br>Organisational culture that is not supportive<br><br>No activation of policies<br><br>Absence of caregiver<br><br>Complicated cases<br><br>Patients refusing counselling<br><br>Prescription being unclear<br><br>Inability to communicate with physician<br><br>Shortage of medications | *"There is one idea that we want to apply in the pharmacy that ...umm...maybe print QR code [...] the one who cannot see will just use the QR code and it will give him the instructions for this medicine"* (HP01)<br><br>*"One time we started a project which was Braille […] So they brought it so that printing could be in Braille, but the project did not work because of some problems."* (HP11)<br><br>*"I would try…I would try as much as possible if the packs were similar, two different medications with similar packs, I'd try to look for an alternative with a smaller pack for example…so that they are different in size."* (CP03)<br><br>*"As I told you, time pressure. No…umm no enough support from…from the organisation […] and…umm and the culture of the…of the organisation, if it's a culture that is very helpful…it will help. If it's a culture that doesn't support you…"* (CP06)<br><br>*"If the case is somewhat severe, definitely no. I'd certainly be afraid. I would let him know that, well, this is a case for the hospital." (CP03)* |

**Determinants (facilitators and barriers) of pharmacists' medication dispensing and counselling behaviour**

| TDF Domain | Facilitators | Barriers | Illustrative quotes |
|---|---|---|---|
| **Intentions (*cont'd*)** | | | *"I mean some of them, already when you try to approach them, he will give you the sign that "Nope, nope, thank you. Sorry, I understand, I know.", or for example "No need, I, like, have someone at home who can take care of me. So, like, there is no need". So, it would be…so you feel, like, even if you…if you say anything, it will be useless. I mean even the patient, he will lose interest, indifferent to you."* (HP10)<br><br>*"The only thing that would prevent us is being unable…the ability to communicate with the physician."* (CP08) |
| **Goals** | VI patients a priority because of their special needs and being more prone to medication problems<br><br>Beliefs about proper dispensing/counselling being a patient right<br><br>Patients/caregivers understanding instructions, patients taking medication properly and coping well during follow-up visits, case less complicated (because pharmacist can then focus on other patients in need)<br><br>Patient having professional help at home (e.g. nurse) (because pharmacist can then focus on other patients in need) | Practice setting (VI patients not coming frequently)<br><br>Time constraints | *"Yes! Prio...prio…priority, the highest level of priority. […] Why? Because this is a living example of people who may take medicine in a wrong way, who may have medication errors at a very high rate, at 200% not 100%...soo, high priority."* (HP04)<br><br>*"For me, not a very high priority. Because I…I mean, these kind of patients do not come frequently to the pharmacy. That is it. So…this can be improved in hospitals, more of in institutions, I mean….or the hospital pharmacy, it would of course be better in this regard. Of course they'd consider it a priority. But in a community pharmacy, that would be a little difficult because there are many community pharmacies and these kind of patients may not be that many considering the spread of pharmacies…"* (CP03)<br><br>*"It depends on how many patients you will see. If you see let's say one patient per year of umm visually impaired patients, ok so this is only one patient. If you see…if you are in an institution umm where you probably see many patients, ok, visually impaired patients, then in this case umm, ok, …there should be training, there should be… using all tools that might…that might help."* (HP06)<br><br>*"If I am sure that they are taking their medications on time, they are understanding my instructions, they are doing ok with their follow-up visits, I wouldn't change anything."* (HP06)<br><br>*"Sometimes too many patients requiring services at the same time …it forces you to be brief in explanation, especially if the patient has been using the medication itself for years."* (HP09) |

| Determinants (facilitators and barriers) of pharmacists' medication dispensing and counselling behaviour | | | |
|---|---|---|---|
| **TDF Domain** | **Facilitators** | **Barriers** | **Illustrative quotes** |
| **Goals (*cont'd*)** | | | *"Less important if…he has for example…a nurse at home or I mean someone who is a healthcare provider at home. Therefore, it would be…it would be, like, easy with them. So the importance is not…the responsibility would not lie only with the pharmacist I mean, there would be someone helping."* (CP06) |
| **MADP** | A note about VI in medical chart<br><br>Patient presenting at the pharmacy in person<br><br>Patients presenting cards indicating their disability<br><br>Decisions based on understanding patients' needs<br><br>Checking the prescription, talking with the patient, reading doctor notes<br><br>Asking about medication quantity at home to assess patient use of medication<br><br>Being aware of prior errors/being sent emails about them/having them presented by the medication safety officer<br><br>Alerts for certain issues, e.g. allergies, duplications | Not everyone reading notes in the medical chart because of time constraints<br><br>Not recognising the patient or not knowing he needs special assistance<br><br>Patient not disclosing VI, pharmacist avoiding asking about VI<br><br>Lack of computer alerts about VI<br><br>Too many patients requiring services at the same time, workload<br><br>Remembering to address issues "by chance"<br><br>Insufficient information about the patient<br><br>Long periods of time between patient's visits<br><br>Focusing on the caregiver | *"Maybe, maybe you can find it* [information about VI] *in the notes. [...] We do open it, but not always, to be honest."* (HP15)<br><br>*"Maybe if someone else comes to dispense, not the patient, you wouldn't know that the patient you dispense for is blind."* (HP10)<br><br>*"As I am telling you, even when they…when he comes into the pharmacy, he never tells that he is visually impaired [...] and of course I never try to say like [...] "Oh, you have an impaired vision?" …he'd consider it like…an insult or something like that."* (CP04)<br><br>*"So, all of this will be umm… be also based on the needs of every person…I mean, it will be tailored to the patient I mean, should not be equal"* (CP01)<br><br>*"There is an alert for duplications. But like the problem is that we somewhat ignore the system's alert because it pops up a lot."* (HP17)<br><br>*"I mean by just remembering the errors that happened or the stories, the history I've been told. [...] Sometimes it is presented to us. [...] The medication safety officer would come and say so. Sometimes [...] you get an email with a full explanation of the issue. So that is like an alert, you need to stop and check."* (HP16)<br><br>*"I give all the directions. But we… for a patient with eye problems, I don't focus closely because as I am telling you I trust that the caregiver will be the one giving him* [the medication]*."* (HP14) |

| Determinants (facilitators and barriers) of pharmacists' medication dispensing and counselling behaviour | | | |
|---|---|---|---|
| **TDF Domain** | **Facilitators** | **Barriers** | **Illustrative quotes** |
| **ECR** | Guidelines (internal guidelines and instructions from administration would help until there are official guidelines)<br><br>Pharmacy design & layout (e.g. dedicated window for patients with a disability)<br><br>Courses on how to communicate with disabled people<br><br>Tools, e.g. brochures, video/sound clips, pill organisers, coloured markers, methods to differentiate between packs<br><br>Large font or Braille labels/instructions useful but may not be practical<br><br>Braille writing on packs offered by some companies<br><br>Checklist with a question about disability and one to help with counselling | Workforce shortage<br><br>Time constraints<br><br>Pharmacy layout<br><br>Relying on technicians because of pharmacist shortage<br><br>Poor resources<br><br>Practice setting (few numbers of VI patients)<br><br>Braille information may be only useful for younger patients who know Braille, not being able to double check braille labels<br><br>Not having access to physicians<br><br>Similar appearance of medications<br><br>Changing medication brands which leads to patient confusion | *"I mean, everyone should not act as they please. These guidelines would give you the final conclusion that they have arrived at using approved and proper medical methods."* (CP03)<br><br>*"I wish there would be a course adopted from universities abroad that deal with this, make it in Arabic and take it… it would be for colleagues who deal with them..."* (HP09)<br><br>*"Also, providing aids, I mean if there were brochures or video clips or sound, sound clips so that he'd listen to sound clips in one way or other. If there is like…some pharmaceutical accessories like organisers…pill organisers and such..."* (HP04)<br><br>*"I'd tell him "Record, no problem, record a video of me* [explaining]*, no problem.""* (HP05)<br><br>*"I would try as much as possible if the packs were similar, two different medications with similar packs, I'd try to look for an alternative with a smaller pack for example…so that they are different in size."* (CP03)<br><br>*"The problems are that it only prints in Braille, it does not print the other* [regular] *text. So, we cannot know…cannot double check, cannot know what was printed. […] So, it stopped until they can fix this problem."* (HP11)<br><br>*"Unfortunately, no, because that is a stan…standard label. Umm we have certain font size, we can't exceed it because it will be excluded…it will be print* [sic] *out of that label, yeah."* (HP08)<br><br>*"...When the pharmacist is providing counselling, there should be like a checklist available to help him with providing the counselling."* (HP04)<br><br>*"Work environment is not, because I have my own clinic, so it's not really big deal…I can just spend* [as] *much time, as much I can spend."* (HP06) |

| Determinants (facilitators and barriers) of pharmacists' medication dispensing and counselling behaviour | | | |
|---|---|---|---|
| **TDF Domain** | **Facilitators** | **Barriers** | **Illustrative quotes** |
| **ECR (*cont'd*)** | Companies manufacturing drugs in different looks/shapes<br><br>Smart phone applications/ QR codes<br><br>Pharmacist practising in a clinic (e.g. clinical pharmacist)/private area for counselling<br><br>Tools to be required and monitored by official bodies<br><br>Patient support services (e.g. clinic for support of blind patients, volunteers to take patients to the pharmacy)<br><br>Methods of identifying that the patient is visually impaired (e.g. disability card, computer alert, note from physician)<br><br>System for recording errors | | *"He sometimes has a card, a disability card. [...] I'd serve him quickly"* (HP09)<br><br>*"... Too many patients requiring services and lack of time, I'm telling you the daily workload ranges from 550 and up to 900 patients divided between eight windows."* (HP12)<br><br>*"I think that there is more interest there than we have here. [...] because the hospital is dedicated to eyes. Here, it is mixed, general with eyes with…"* (HP14)<br><br>*"...One of the obstacles we face with these things is poor resources. There is no support. [...] Because as I've told you, the administration has no knowledge of what you are doing in the pharmacy. ''All you have is a prescription, you're at the window [dispensing] and that is it''. They know nothing about these other things."* (HP04)<br><br>*"It is very difficult, impossible to reach the doctor there [...] even at private clinics it would be hard to reach him. He is with patients the whole time because of the short operation hours."* (CP04)<br><br>*"...Sometimes the medicine's company changes. I mean previously he was used to the medication and knows its shape and texture, so it would be difficult to convince him that this is the same medication but from a different company..."* (HP09) |

| Determinants (facilitators and barriers) of pharmacists’ medication dispensing and counselling behaviour | | | |
|---|---|---|---|
| **TDF Domain** | **Facilitators** | **Barriers** | **Illustrative quotes** |
| **Social influences** | Patients<br><br>Caregivers<br><br>Physicians/nurses<br><br>Pharmacy colleagues<br><br>Social workers<br><br>Senior management | Patients not having confidence in pharmacist or refusing counselling<br><br>Caregivers<br><br>Other patients<br><br>Physicians<br><br>Pharmacy colleagues<br><br>Pharmacy trainees<br><br>Senior management | *"I mean there are patients that you feel like they are very careful about themselves, you feel they have the willingness “Yes, tell me. Yes, I want to see…”and some would tell you like “Just finish. I’ll take the medication and leave. It is none of your business. Don’t...” I mean it depends on the attitude of the patient."* (HP03)<br><br>*"The patient sometimes has more confidence in the physician than the pharmacist, he does not get convinced with what the pharmacist is telling him or does not appreciate the importance of the pharmacist."* (HP04)<br><br>*"Here, when we dispense for those with special needs, other patients jump in “why did you let them go* [first]*”. [...] I mean, he is barely concentrating with me, and this one comes to interrupt me and him. So, my train of thought…you must go through it all over again."* (HP15)<br><br>*"Umm… usually… umm actually nobody except for probably the caregiver, if they have a caregiver who can really help. [...] if… the caregiver cannot really deal with this, this is difficult because sometimes you rely on them, if you don’t have any other tools to…."* (HP06)<br><br>*"...I’d talk to the nurse and communicate directly with the physician and explain to him that this thing is wrong. [...] Explain to him I mean. And the vast majority are fine and responsive. I mean we never struggle with this."* (HP13)<br><br>*"I’d notify him about our intervention and the issue of the medication error, but he wouldn’t not accept this. Because “Nope, I’m the physician and what do you know! Do as I say” and this has consequences and it happens."* (HP04)<br><br>*“I may be influenced by the rest of my colleagues who are not working or making an effort. And at the end we are treated equally. [...] on the long run, it might influence me."* (HP11)<br><br>*"Because there are students so when they return the medication, they return it wrong. [...] there were 2 mg [strips] placed there by mistake"* (HP10) |

| Determinants (facilitators and barriers) of pharmacists' medication dispensing and counselling behaviour | | | |
|---|---|---|---|
| **TDF Domain** | **Facilitators** | **Barriers** | **Illustrative quotes** |
| **Social influences (*cont'd*)** | | | *"If they included something about the staff being supposed to cover this aspect [through] for example a lecture per year, safety…as part of safety. Umm a protocol or something in which each employee is required to attend at least one* [sic] *per year this um...so he would understand and be qualified."* (HP14)<br><br>*"Basically, even the supervisor himself wants you to, like, finish patients faster…don't say…just finish quickly…don't say…give a lot of information, just give the basics and finish."* (HP15) |
| **Emotion** | Feeling satisfied/happy/ proud with preforming duty and helping patients<br><br>Sense of achievement<br><br>Feeling greater responsibility<br><br>Sympathy as positive influence<br><br>Obsession with providing full service to clear conscience | Stress may affect quality of service<br><br>Fear of: making mistakes, patient having multiple disabilities, patient coming alone<br><br>Feeling greater responsibility (wishing others would deal with patient) | *"Of course, I feel proud that I umm was able to contribute to improving his condition, that he would be always safe taking his medicine."* (CP06)<br><br>*"My work stress would really reduce the quality of my work, and my sympathy for the patient would improve my performance to…to a high degree. Of course, if I were emotionally engaged, I might really not sleep unless I find a certain way."* (HP03)<br><br>*"...But the pressure would be a little higher than with the regular patient. You'd be afraid um he'd make a mistake and you'd be the cause. This fear or the… the…this is present with every patient, but it increases with special cases.* (HP12)<br><br>*"I feel like what I've been entrusted with has increased. You get it? That…it honestly is a responsibility. I mean you'd wish that these people, [...] like, to have the supervisor to deal with them, maybe especially the blind, or something like that. Because I don't know, like sometimes you'd wish to relieve yourself of this responsibility. You get it? Sometimes, I mean you are just afraid."* (HP03) |
| **Behavioural regulation** | Checklist to help with providing counselling<br><br>Asking patient about medication & previous counselling<br><br>Asking patient to repeat instructions | No method to measure effect of counselling<br><br>Lack of formal policies/procedures<br><br>Pharmacist documentation not always done | *"This is something we need during counselling…when the pharmacist is providing counselling, there should be like a checklist available to help him with providing the counselling."* (HP04)<br><br>*"I would ask him to repeat the sentence I have told him, the medications use instructions. [...] so I ask him to repeat the explanation and explain it to me again. If there is any question,… When he says everything is ok, that is it, thank God."* (HP13)) |

| Determinants (facilitators and barriers) of pharmacists' medication dispensing and counselling behaviour | | | |
|---|---|---|---|
| **TDF Domain** | **Facilitators** | **Barriers** | **Illustrative quotes** |
| **Behavioural regulation (*cont'd*)** | Following-up with patients/ caregivers (feedback)<br><br>Pharmacist documentation<br><br>Experience<br><br>Patient coming before refill time<br><br>Patient outcomes<br><br>Code of conduct<br><br>Medication error report<br><br>Key performance indicators (KPIs) | | *"Maybe at the next dispensing…sometimes, he'd come to you before the medication finishes and tells you "I've finished it!" Ok, how did you finish it and you are supposed to still have stock? That is evidence that he either lost it, or is, like, taking it in a wrong way."* (HP09)<br><br>*"I mean for example I have a report for medication errors. Whenever either a near miss or any other error occurs, we all record it. So that is an indicator um that would let us know what happened.* (HP14)<br><br>*"But now we, we, with the…with the KPIs and these things, you know, the key performance indicators, there might be something that can assess. It might be a questionnaire for patients."* (HP14)<br><br>*" I mean it is mostly personal efforts, there is no guideline for us to follow."* (HP14)<br><br>*"The problem we have is that we do not have things to measure the…our productivity […] the … instructions, all of these things that you do, did it affect the patient? Did it make the patient better?"* (HP01) |

***Abbreviations:*** CP= Community Pharmacist; ECR= Environmental context and resources; HCPs= Healthcare professionals; HP= Hospital pharmacist; KPI= Key performance indicator; MADP= Memory, attention, and decision processes; QR code= Quick response code; SPRI= Social/professional role and identity; VI= Vision impairment
